# Femoral Stems With Porous Lattice Structures: A Review

**DOI:** 10.3389/fbioe.2021.772539

**Published:** 2021-11-17

**Authors:** Bolun Liu, Huizhi Wang, Ningze Zhang, Min Zhang, Cheng-Kung Cheng

**Affiliations:** ^1^ Key Laboratory of Biomechanics and Mechanobiology, Ministry of Education, Beijing Advanced Innovation Center for Biomedical Engineering, School of Biological Science and Medical Engineering, Beihang University, Beijing, China; ^2^ School of Biomedical Engineering, Shanghai Jiao Tong University, Shanghai, China

**Keywords:** femoral stem, porous structure, stress shielding, bone ingrowth, additive manufacturing, total hip arthroplasty

## Abstract

Cementless femoral stems are prone to stress shielding of the femoral bone, which is caused by a mismatch in stiffness between the femoral stem and femur. This can cause bone resorption and resultant loosening of the implant. It is possible to reduce the stress shielding by using a femoral stem with porous structures and lower stiffness. A porous structure also provides a secondary function of allowing bone ingrowth, thus improving the long-term stability of the prosthesis. Furthermore, due to the advent of additive manufacturing (AM) technology, it is possible to fabricate femoral stems with internal porous lattices. Several review articles have discussed porous structures, mainly focusing on the geometric design, mechanical properties and influence on bone ingrowth. However, the safety and effectiveness of porous femoral stems depend not only on the characteristic of porous structure but also on the macro design of the femoral stem; for example, the distribution of the porous structure, the stem geometric shape, the material, and the manufacturing process. This review focuses on porous femoral stems, including the porous structure, macro geometric design of the stem, performance evaluation, research methods used for designing and evaluating the femoral stems, materials and manufacturing techniques. In addition, this review will evaluate whether porous femoral stems can reduce stress shielding and increase bone ingrowth, in addition to analyzing their shortcomings and related risks and providing ideas for potential design improvements.

## Introduction

Total hip arthroplasty (THA) is a surgical procedure that replaces the diseased joint with an artificial femoral stem and acetabular cup. It can effectively relieve pain, restore joint function, and correct deformities of the hip joint. This procedure has become one of the most successful surgical interventions in the past century for improving quality of life. It has been reported that approximately 400,000 THA procedures are performed in China every year, with an annual growth rate of 25–30% (Dai et al., 2015). Implant loosening is the most common complication that requires revision surgery (Slif D. Ulrich et al., 2008; Kärrholm et al., 2016; National Joint Replacement Registry A.O.A., 2017).

The main mechanical factor that causes loosening of cementless hip prostheses is stress shielding. Naturally, when a load is applied to the intact femoral head, stress is transmitted through the trabecular bone of the femoral neck to the cortical bone of the proximal femur. When the femoral component of hip replacement was inserted into the medullary cavity, the prosthetic femoral stem and the remaining bone of the femur constitute a new stress transmission system due to the mismatched properties. The current femoral stems on the market are mostly made of dense metal, such as titanium-based alloy, cobalt-chromium alloy, or 316L stainless steel, with a Young’s modulus (110–230 GPa) far greater than that of bone (0.3–22 GPa) (Pałka and Pokrowiecki, 2018). Thus, the much stiffer metal implant will bear most of the load while the bone surrounding the implant will have a greatly reduced stress. Bone remodeling is a dynamic process affected by mechanical stimulation which leads to bone formation under high load and bone resorption under low load (Wolff, 2010). Stress shielding can lead to bone resorption due to the lack of stress stimulation on the proximal femur. Bone resorption around the implant also prevents sufficient bone ingrowth into the porous femoral stem, further exacerbating implant loosening (Chen et al., 2009; Kress et al., 2012; Kutzner et al., 2016).

At present, there are two ways to reduce stress shielding. One is to change the stress transmission path by changing the geometry profile of the femoral stem, such as shortening the length of the stem (Bieger et al., 2012; Lerch et al., 2012; Falez et al., 2015), adding a collar to the stem (Jeon et al., 2011; Rami M. A.; Al-Dirini et al., 2017), or matching the geometry with the proximal femoral canal (Ostbyhaug et al., 2009; Hua et al., 2010). However, the initial stability (Giardina et al., 2018) and alignment accuracy (Khanuja et al., 2014; Shishido et al., 2018) of short stems is still questionable, and collars are only mechanically effective when there is good contact with the calcar (Jeon et al., 2011). The other way is to reduce the integral stiffness of the femoral stem, such as by adopting an internal hollow structure (Gross and Abel, 2001; Yang et al., 2009), lower stiffness composite structure (Glassman et al., 2001; Hartzband et al., 2010), grooves (Wu et al., 2018; Heyland et al., 2019), slotted design on the distal end (Cameron, 1993), or using a metallic porous structure (Limmahakhun et al., 2017a; Arabnejad et al., 2017; Jette et al., 2018; Wang et al., 2018). Given that most bone resorption occurs around the proximal femur, reducing the stiffness of the distal end by using a grooved or slotted end design does little to reduce stress shielding (Glassman et al., 2006). A composite stem with a solid cobalt-chromium core surrounded by a layer of porous PEEK coating has the potential to reduce stress shielding. The porous PEEK coating is biocompatible and chemically stable, and has a stochastic matrix structure with an average pore size of 300 µm (Nieminen et al., 2007; Ma and Tang, 2014). It is also reported that PEEK coating was dissociated from the central core, leading to periprosthetic infection and loosening (Saltzman et al., 2014). Femoral stems with an internal hollow structure are a viable alternative but are difficult to fabricate with traditional subtractive manufacturing.

In addition to bone resorption from stress shielding, the long-term secure fixation of cementless femoral stems is dependent on the growth of bone into or onto the rough surface of the stem (Sporer and Paprosky, 2005). This osseointegration provides biological fixation and secondary stability for the femoral stem, enhancing load transfer from the stem to the bone and decreasing stress shielding (Bobyn et al., 1987). Surface coatings, such as sintered beads, hydroxyapatite coating, grit-blasted surfaces, and titanium plasma spray have been used in orthopedic implants over the past 40 years to promote osseointegration. However, two of the major shortcomings of these coatings are an insufficient adherence to the substrate and a non-uniform thickness, which can leave some regions with a thin surface layer or low porosity, leading to poor bone ingrowth (Murr et al., 2010).

With the advent of additive manufacturing (AM) technology (also known as 3D printing) it is now possible to fabricate femoral stem with internal porous structure. As expected, porous metal has a lower stiffness than a solid implant (Cheng et al., 2012), and thus varying the quantity, size and location of the pores can be used to tailor the stiffness of the femoral stem and reduce stress shielding around the proximal femur (Arabnejad et al., 2017; Wang et al., 2018; Mehboob et al., 2020a). Moreover, incorporating open and interconnected pores in an implant can provide space for the transportation of nutrients and the ingrowth of bone tissue, thereby increasing the implant’s secondary stability (Taniguchi et al., 2016; Wang et al., 2016).

Given the potential benefits of these novel designs, research is ongoing and is producing encouraging results. Studies have already investigated the use of porous structures in orthopedic implants, such as the improved mechanical properties of a lattice cube design (Amin Yavari et al., 2013; Gómez et al., 2016) and the effect of pore morphology on bone ingrowth behavior (Wu et al., 2013; Taniguchi et al., 2016). Other review articles concentrated on the porous structure, focusing on geometric modeling, design parameters, manufacturing techniques, mechanical properties, and potential applications (Babaie and Bhaduri, 2017; Savio et al., 2018). However, it is not enough to study porous structure alone, the geometry of the stem body and distribution of the porous structure can also have a considerable impact on the safety and effectiveness of the femoral stem. Therefore, this article aims to present a comprehensive review and discussion on porous femoral stems.

This review will focus on the latest research into porous femoral stems, including different types of porous structures, the macro geometry, mechanical function, research methods used during development, materials and manufacturing techniques. This review also includes studies on the ability to reduce stress shielding and promote bone ingrowth. The intent is to provide an informative reference for the development of porous femoral stems.

## Review Method

This review was conducted following the Preferred Reporting Items for Systematic Reviews and Meta-Analyses (PRISMA) guidelines.

### Literature Search Strategy

A comprehensive literature search was carried out by using the Web of Science and PubMed databases, and all the concerned English publications up to May 2021 were collected. The literature search strategy was that A search with keywords “femoral stem” or “hip stem” was first performed. In the selected published literatures, the papers including either “porous” or “porous structure” or “cellular structure” or “lattice structure” or “biomimetic” or “metallic foams” were collected.

### Study Selection: Inclusion Criteria and Quality Assessment

After removing duplicates, the returned articles were filtered through two stages. The first stage was to conduct a preliminary screening by title and abstract. If the item was not a research article, such as review articles or conference abstracts, they were excluded. Papers that were not related to femoral stems with a porous internal structure were also excluded at this stage, such as porous coatings, tissue engineering, acetabular cups, other joint implants, etc. According to the above exclusion criteria, the quality assessment was carried out by screening the articles’ full text in the first stage. Two independent reviewers conducted the data extraction, and an additional reviewer judged disagreements between two reviewers.

**FIGURE 1:**
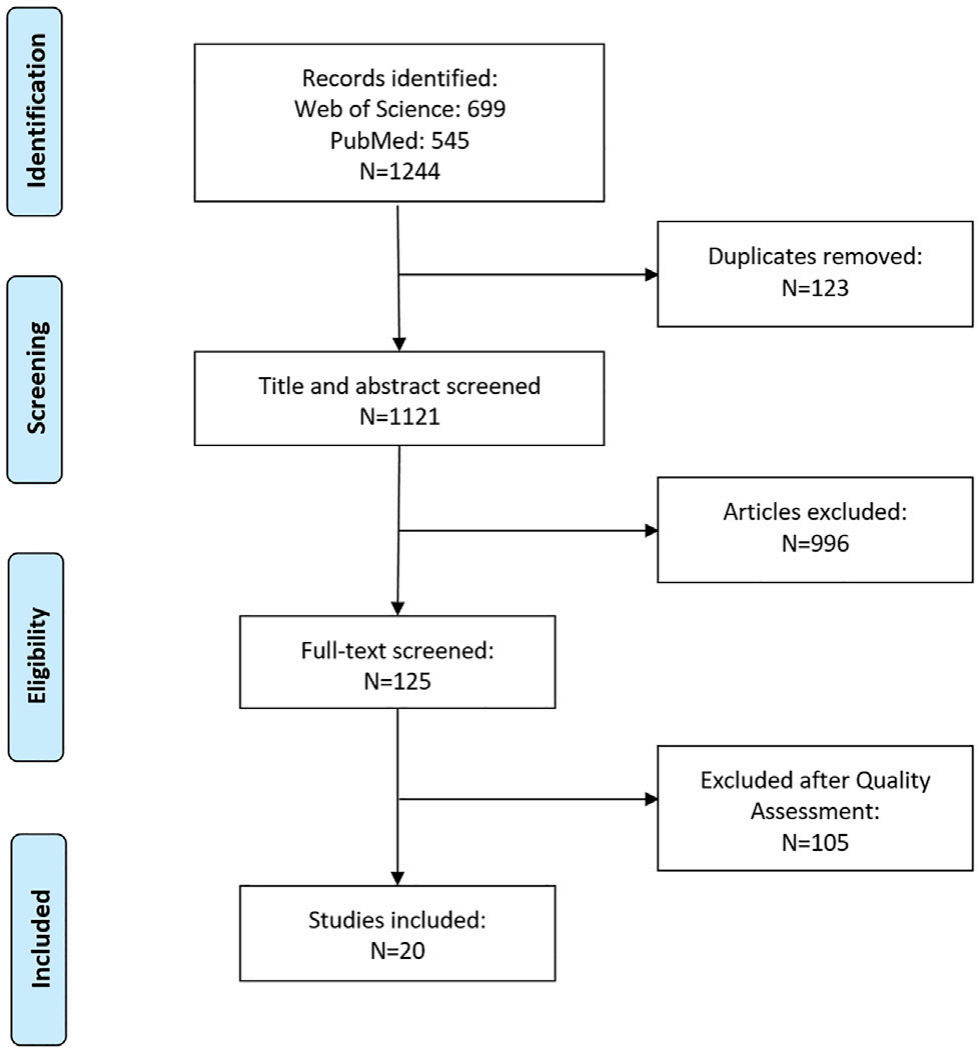
Flow diagram of search strategy.

A total of 1,244 articles were identified, of which 699 were retrieved from Web of Science and 545 from PubMed. After excluding 123 duplicates, 1,121 articles were screened based on their title and abstract, and 125 were selected for full-text assessment. After excluding articles based on assessment criteria, 20 articles were included in this review. A flow diagram showing the search strategy is presented in Figure 1.

## Porous Structures Applied to the Femoral Stem

When designing a porous structure for the femoral stem, the design can be influenced by mechanical factors and the need for biological adaptation. The former is characterized primarily by stiffness, while the latter mainly considers the pore size and porosity.

The stiffness of porous structures can be expressed by the Young’s modulus, which is determined by a stress-strain curve according to Hook’s law, as shown in Eq. 1.

$$E_{effective}=\frac{\sigma }{\varepsilon }=\frac{\frac{F}{A}}{\frac{L}{\Delta L}}$$ (1)

Where σ is the stress, ε is the strain, F is the force acting on the porous unit cell, A is the cross-sectional area of the porous unit cell, and L and ∆L are the original length and the change in length of the porous structure respectively.

The closer the stiffness of the femoral stem material is to femoral bone (15–25 GPa) (Limmahakhun et al., 2017a), the lesser the effect of stress shielding (Heinl et al., 2008). The stiffness of porous structures is related to porosity or relative density. According to Gibson and Ashby (Gibson and Ashby, 1988), the relationship between the equivalent Young’s modulus and relative density can be expressed as Eq. 2.

$$\frac{E}{E_{s}}=C{\left(\frac{\rho }{\rho_{s}}\right)}^{n}$$ (2)

Where E is the elastic modulus of the porous material, E_s_ is the elastic modulus of the solid material, and C and n are constants that depend on the porous structure. The constant of C for porous structures depends on unit cell type, ranging from 0.1 to 4.0. Typically, n is a constant varying from 1 to 3. And ρ and ρ_s_ are the density of the unit cell and the density of the constitutive material, respectively. The porosity φ can be defined by Eq. 3. Higher values of φ indicate more space for bone tissue growth but lower strength. Porosity should be at least 50% to promote adequate bone ingrowth (Arabnejad et al., 2016).

$$\phi =1-\frac{\rho }{\rho_{s}}=\frac{V_{voids}}{V_{total}}$$ (3)

Where V_voids_ is the volume of the voids and V_total_ is the volume of the entire porous structure. Also, the pore size is an important factor affecting bone ingrowth (Dhiman et al., 2019). Pore size is often defined as the largest inscribed circle in a unit cell of the porous structure. Due to ethical constraints, the pore size suitable for bone ingrowth is mainly assessed by animal *in vivo* experiments. At present, femoral condyle of rabbits (Taniguchi et al., 2016), femur of rats (Van der Stok et al., 2013), metatarsus of goats (Li et al., 2016), acetabular and femur of canines (Bobyn et al., 1980; Jasty et al., 1989) have been used as experiment subjects. Different experimental subjects and bone types present different suitable pore sizes for bone ingrowth. The reported range for bone ingrowth is within 50–800 μm, and this range is typically used to guide the design of porous structures for the femoral stem (Harrysson et al., 2008; Arabnejad et al., 2016).

The design of the porous structure can be characterized as either a regular structure (unit cell structure) or stochastic structure (irregular structure). This section will focus on the structural and mechanical properties of these structure types and assess the advantages and disadvantages of each.

### Regular Structure

Most porous femoral stems use a regular structure type, with the key advantage being that it is easy to tailor the mechanical properties by adjusting the design parameters. The pore size and porosity can be manipulated to promote bone ingrowth.

**FIGURE 2:**
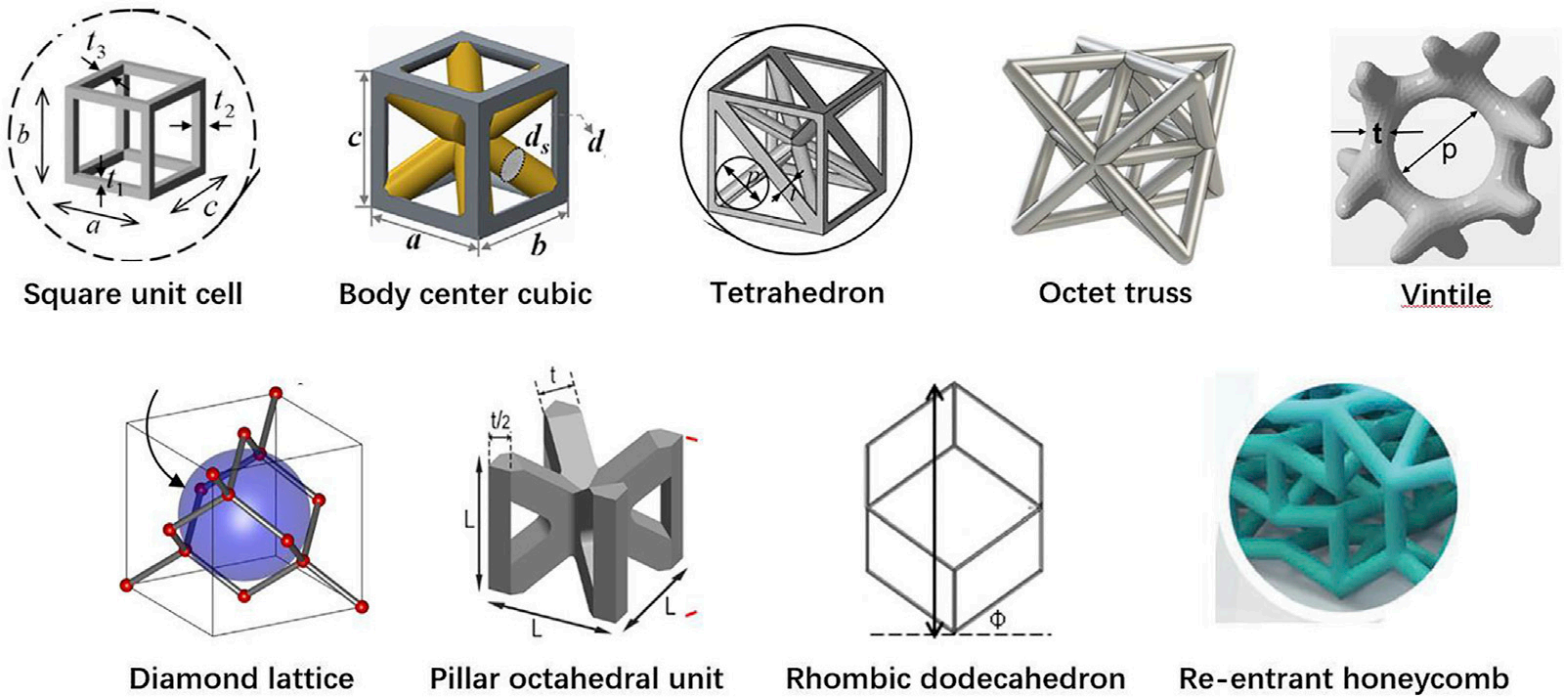
Regular porous structures used in the design of femoral stems.

Among the 20 pieces of literature included in the study, most used regular porous structures; square unit cell, body center cubic, tetrahedron, octet truss, vintile, diamond lattice, pillar octahedral unit, rhombic dodecahedron, and re-entrant honeycomb. The regular porous structures reviewed in this article are summarized in Figure 2. Table 1 is a comprehensive summary of porous structures applied to femoral stems from literature. It exhibited the geometric features and mechanical properties of each regular porous structure used for femoral stems in the collected literatures.

**TABLE 1:** Geometric features and mechanical properties of different regular porous structures applied to femoral stems.

Unit cell	Material	Porosity (%)	Unit cell size (mm)	Pore size (mm)	Strut thickness (mm)	Young’s modulus (Gpa)	Yield strength (MPa)	Ultimate compressive strength (MPa)	References
**Square unit cell**	Ti6AL4V	57–64	-	0.6–3.24	1.1–3.08	9.27–4.74	134	-	Eldesouky et al. (2017b)
	Ti6AL4V	57	-	0.6	1.1	9.27	197	-	Eldesouky et al. (2017a)
	Ti6AL4V	89.6–21.6	4	-	0.3–0.9	4.86–71.36	-	-	Mehboob et al. (2018)
	Ti6AL4V	10–100	1.36–1.8		0.07–0.1				Arabnejad and Pasini, (2012)
	Ti6AL4V	> 40	-	0.05–0.8	> 0.1	-	-	-	Arabnejad Khanoki and Pasini, (2013)
	CoCrMo	-	-	1.57–1.88	0.5–1.5	4.79–17.98	65.4–295.7	-	Hazlehurst et al. (2014a)
	CoCrMo	-	-	1.57–1.88	0.5–1.5	4.79–17.98	65.4–295.7	-	Hazlehurst et al. (2014b)
**Body center cubic**	Ti6AL4V	20–80%	4	-	1.25–0.51	8–70	-	-	Alkhatib et al. (2019b)
	Ti6AL4V	78.7–29.3	4	-	0.3–0.9	18–79	188–839	-	Mehboob et al. (2018)
	Ti6AL4V	90–18	-	-	0.33–1.25	2.8–76.7	37–760	-	Mehboob et al. (2020b)
**Tetrahedral**	Ti6AL4V	-	-	-	-	-	-	-	Arabnejad et al. (2017)
	Ti6AL4V	-	-	0.05–0.8	> 0.2	-	-	-	Wang et al. (2018)
**Diamond lattice**	Ti6AL4V	89.4–21.5	4	-	0.3–0.9	0.55–54.18	-	-	Mehboob et al. (2018)
	Ti6AL4V	58	-	0.8	0.54	8.4	91	-	Jette et al. (2018)
	Ti6AL4V	60–40	-	0.05–0.8	> 0.3	7.3–12.1	136.7–274.5	-	Wang et al. (2020)
**Pillar octahedral unit**	CoCrMo	67–14	-	-	-	2.33–5.26	36–299	113–916	Limmahakhun et al. (2017a)
**Rhombic dodecahedron**	Ti6AL4V	59–96	3–12	-	-	-	-	85.7–0.85	Harrysson et al. (2008)
**Vintile**	Ti6AL4V	40–58.5	4–7	0.8–1.5	0.7–1.5	0.68–1.58		1,431–5,021	Abate et al. (2019)
**Re-entrant honeycomb**	Ti6AL4V		-	-	0.35	34–43	-	≈2.4	Kolken et al. (2018)
**Honeycomb**	Ti6AL4V		-	-	0.35	38–50	-	≈2.4	Kolken et al. (2018)

**FIGURE 3:**
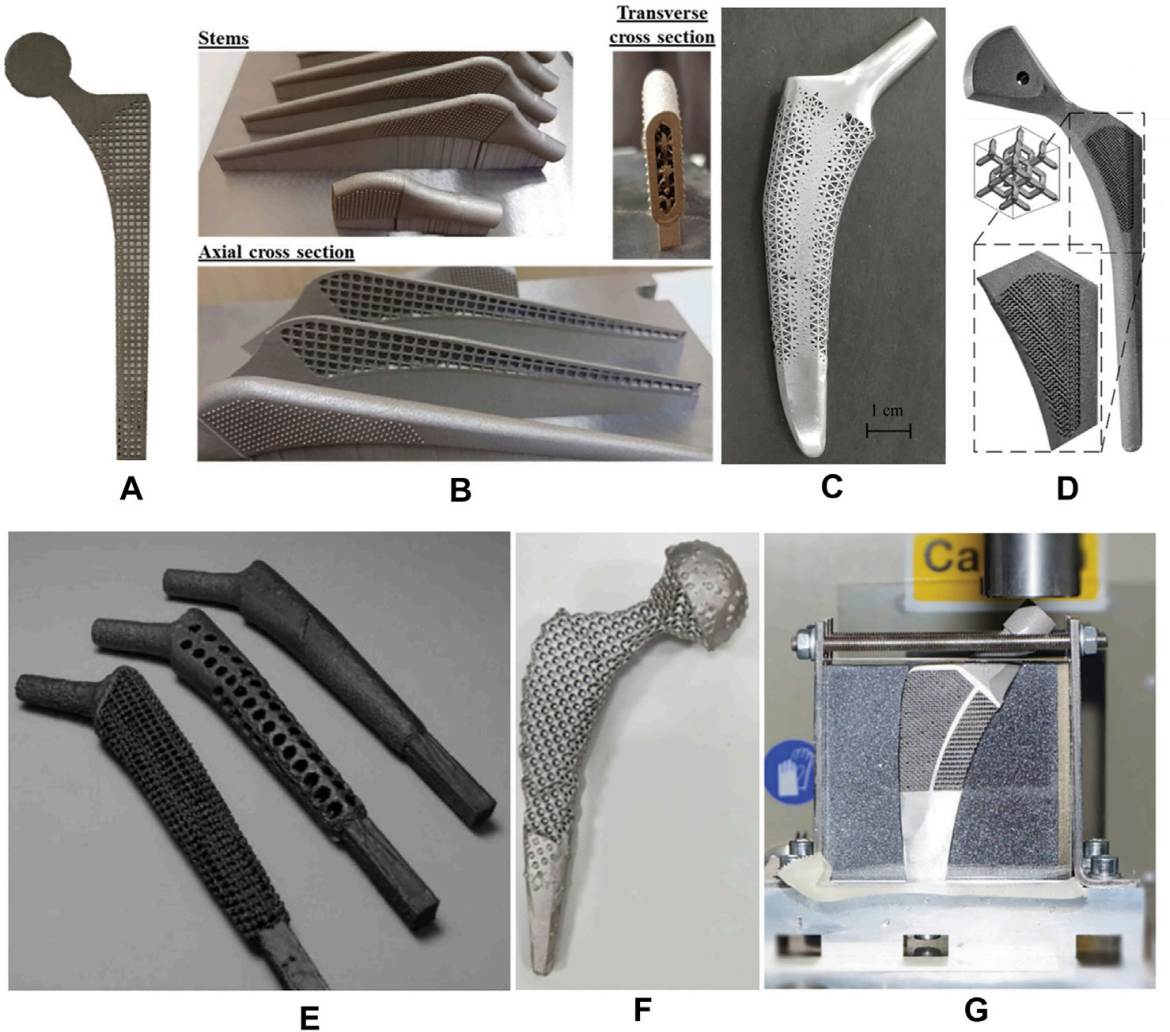
Femoral stem with regular porous structure. **(A)** 2D femoral stem using square unit cells designed by Arabnejad Khanoki and Pasini (2013); **(B)** femoral stem using BCC unit cells designed by Mehboob et al. (2018) **(C)** femoral stem using tetrahedral topology designed by Wang et al. (2018); **(D)** Stem using diamond lattice unit cells designed by Jette et al. (2018); **(E)** Stem using Rhombic dodecahedron unit cells designed by Harrysson et al. (2008) **(F)** Stem using vintile lattice unit cells by Abate et al.; (Abate et al., 2019; Abate et al., 2021); **(G)** Meta stem designed by Kolken et al. (2018), with the lateral part of the femoral stem having a re-entrant honeycomb structure and the medial part having a honeycomb structure.

Of the 20 articles included in this study, the square unit cell, also known as cubic unit cell, appeared in six articles (Arabnejad and Pasini, 2012; Arabnejad Khanoki and Pasini, 2013; Hazlehurst et al., 2014a; Hazlehurst et al., 2014b; Eldesouky et al., 2017a; Eldesouky et al., 2017b; Mehboob et al., 2018). This unit cell structure is simple and its mechanical properties are orthogonally symmetric, i.e. the same value of Young’s modulus in the transverse and longitudinal directions (Arabnejad et al., 2017). The cell offers good elastic stiffness along the loading direction (Arabnejad Khanoki and Pasini, 2013), and the axial stiffness can be controlled by adjusting the strut thickness. Mehboob et al. investigated finite element models of cubic, Body Center Cubic (BCC), and diamond structures with the same porosity and applied a compression load to each and found that the cubic cell has the highest Young’s modulus and the highest equivalent yield strength (Mehboob et al., 2018). However, because the struts are prone to bending under shear loading, the stiffness under shear is lower than other unit cells (Egan et al., 2017). In addition, the horizontal struts of the square unit cell are not self-supporting, so it is difficult to fabricate by additive manufacture. Eldesouky et al. overcame this by rotating the printing direction 45° to allow the horizontal struts to be printed without additional support (Eldesouky et al., 2017b). Besides, in an early exploration of this unit cell, Arabnejad et al. fabricated the femoral stem incorporating square unit cells to prove the feasibility of porous femoral stem design (Arabnejad Khanoki and Pasini, 2013) (Figures 3–A).

Another common cell type used in the femoral stem is Body Center Cubic (BCC) (Mehboob et al., 2018; Alkhatib et al., 2019b; Mehboob et al., 2020a; Mehboob et al., 2020b). BCC is similar in design to the square unit cell with the addition of inclined cylindrical struts inside each cell. BBC offers good mechanical properties under axial compression, bending, and torsion loads and exhibits stronger isotropic mechanical properties than the square unit cell. Similarly, Ti6Al4V BCC and Ti6Al4V square unit cell structures have been shown to have comparable effective Young’s moduli and effective yield strengths under axial compression loading (Mehboob et al., 2018). However, under bending and torsion, the effective bending modulus and the effective torsional modulus of BCC is greater than square unit cells. Mehboob et al. developed a femoral stem composed of BCC units combined with an outer dense shell coated with beads to improve the mechanical properties of the stem while providing a porous surface for bone ingrowth (Mehboob et al., 2018) (Figures 3–B).

Femoral stems have also been designed with tetrahedral structures composed of tetrahedral units that have a good capability to restraint tensile and compressive loading, and thus the stability and axial strength of these structures is superior to those composed of bending dominating unit cells (Arabnejad et al., 2017; Wang et al., 2018). Due to the cubic symmetric stiffness matrix of tetrahedral topology, they display almost isotropic mechanical properties (Deshpande et al., 2001). It has been shown that according to the given loading conditions, constraints, and performance indicators, the relative density of each tetrahedral element may be controlled by changing the strut thickness, which can be used to optimize the mechanical properties of different regions of the femoral stem. Wang et al. used boundary representation (B-rep) to represent the geometry of tetrahedral unit cell (Wang et al., 2018) (Figures 3–C). That is, each tetrahedral unit cell geometry is represented by vertices, edges, loops, and faces. Two adjacent tetrahedral unit cells share the same vertex and edge. The relative density of the tetrahedral elements can be adjusted by changing the coordinates of other non-adjacent vertices. Also, because adjacent cells share the same edge and vertex, the geometric transition between cells is relatively smooth, forming a gradient-free topological relationship.

Some studies investigated incorporating more complex diamond lattice structures in the design of femoral stems (Jette et al., 2018; Mehboob et al., 2018; Wang et al., 2020). Compared with square and tetrahedral unit cells, the mechanical properties of the diamond lattice are closer to bone and have a more uniform stress distribution and better isotropic mechanical properties (Quevedo González and Nuño, 2016; Zadpoor and Hedayati, 2016). Also, without horizontal struts, the diamond lattice can be fabricated by additive manufacturing technology without additional supports. In addition, the diamond lattice permits excellent osteogenesis. Taniguchi et al. evaluated the biocompatibility of diamond lattice structures through *in vivo* experiments, showing that a pore size of 600 μm is most suitable for bone ingrowth (Taniguchi et al., 2016). Similarly, Wang et al. reported a suitable pore size of 500 μm (Wang et al., 2019).

Limmahakhun et al. (Limmahakhun et al., 2017a) designed and fabricated a porous femoral stem using CoCr pillar octahedral units. This unit cell is a BCC type without eight horizontal struts, and the horizontal stiffness isotropy is lower than BCC (Lv et al., 2021). Mechanical tests carried out on four cylindrical pillar octahedral specimen with a pore size of 2 mm and porosity ranging from 41 to 67% showed that the mechanical properties of the CoCr components were close to cortical bone, with a Young’s modulus and compressive strength of 2.33–3.14 GPa and 113–523 MPa, respectively. These pillar octahedral structures are also capable of better energy absorption (24.6–116.86 MJ/m3) than bone tissue (Limmahakhun et al., 2017b).

Rhombic dodecahedron unit cells was selected by Harrysson et al. to construct porous femoral stems (Figures 3–E) (Harrysson et al., 2008). Each unit consists of 12 congruent rhombus’ without horizontal struts, and the strut angle to the building plane is 35.26°. Because the electron beam melts the metal powder layer by layer, if the angle between the strut and the building plane is too small, the overlapping area between the layers will be reduced, resulting in a weak structure. Therefore, rhombic dodecahedron structures are suitable for fabrication by Electron Beam Melting (EBM). Harrysson’s study is an early exploration of this type of porous structure that focused on the stiffness of the femoral stem. This study did not assess bone ingrowth, and it should be noted that the cell size used (3–12 mm) is much larger than the suitable pore size (50–800 μm) for bone ingrowth identified in other studies (Harrysson et al., 2008). In addition, although Harrysson’s study considered the strength of the femoral stem and found that high porosity can lead to a significant reduction in compressive strength, the fatigue durability under cyclic loading is also an important factor that should be considered.

In 2019, Abate et al. designed a novel cellular structure termed “vintile,” as shown in Figure 2 (Abate et al., 2019). Compared with common lattice structures, such as cubic and tetrahedron, vintiles have more bearing struts and a smoother transition between struts. This allows for fewer stress concentrations while maintaining excellent structural strength. Abate et al. designed a porous femoral stem using a vintile lattice with porosity ranging from 41 to 71% (Figures 3–F) (Abate et al., 2021). Using mechanical tests and finite element analysis, Abate et al. found that porosities of 56 and 58% resulted in a stiffness of 1.581 and 1.252GPa, respectively, and compressive strength of 5.021 and 4.688GPa, respectively, which is close to that of human bone. While further development work may be required, a vintile structure has shown potential to reduce stress shielding and promote osseointegration in femoral stems.

Kolken et al. designed a femoral stem using a combination of a re-entrant honeycomb structure (Figure 2) and honeycomb structure (Kolken et al., 2018) (Figures 3–G). Different from porous structures described above, a re-entrant honeycomb is a special porous structure with a negative Poisson’s ratio, meaning the whole structure expands radially when placed under axial tension. The negative Poisson’s ratio comes from the deformation and rotation of the structural struts. Kolken et al.’s study did not discuss how the structure allows for bone ingrowth or how the mechanical properties are similar to bone tissue. But aimed to increase the fixation of the femoral stem by expanding the auxetic structure laterally under load, which can theoretically reduce the stress shielding.

In the design of porous femoral stems, in addition to considering the stiffness, strength, isotropy, and potential for bone ingrowth, the arrangement direction and distribution of pores also needs to be considered. In the future, the porous structure might be individually designed and distributed according to the weight, bone density, and morphology of the medullary cavity to achieve the optimal mechanical and biological adaptation characteristics. Another option may be to incorporate different types of unit cells in different areas of the stem body according to the stress distribution of the femoral stem. The mechanical properties could then be tailored to take advantage of the strengths of the various types of porous structures in response to certain loading conditions.

In addition, regular porous cells need to match the geometric profile of the femoral stem. Uneven surfaces or sharp edges may cause difficulty with implantation or produce stress concentrations after implantation. It may be difficult for regular porous unit cell to fit well with the stem curvature or edges, in which case optimizing the structure of the unit cell in the outermost layer for a better adaption to the stem surface may be the solution.

### Stochastic Structure

**FIGURE 4:**
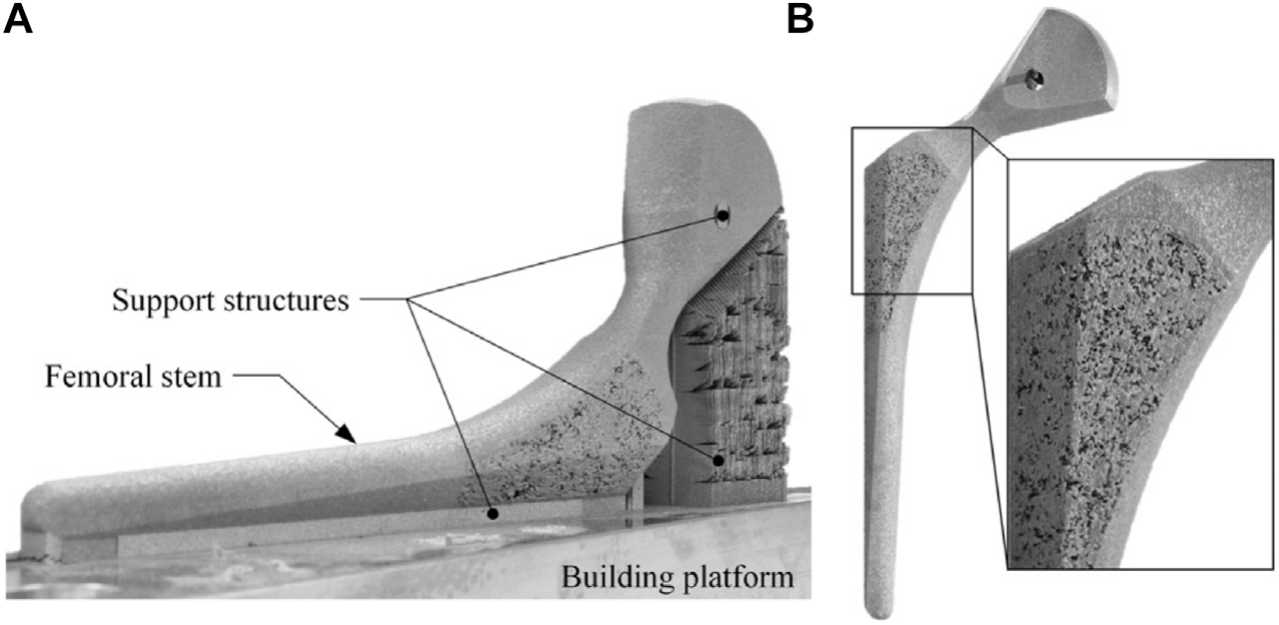
Femoral stem with stochastic porous structure: **(A)** porous femoral stem immediately after selective laser melting processing. **(B)** porous femoral stem after post-treatment, including residual powder removal, separation from the manufacturing platform, and micro-blasting with aluminum oxide (Simoneau et al., 2017).

Compared with regular porous structures, stochastic structures are generally more isotropic (Alkhader and Vural, 2008) and have a microstructure more similar to human cancellous bone. At present, the fabrication of metal stochastic porous structures is mainly through powdered metallurgy (Capek and Vojtěch, 2015) and additive manufacturing. Using additive manufacturing, Simoneau et al. created a femoral stem with a stochastic porous structure composed of interconnected randomly distributed volume pixels (Simoneau et al., 2017) (Figure 4). The mechanical characteristics of such structures can be adjusted by changing either the voxel size or the pore volume fraction (PVF). The former is the side length of the volume pixel, and the latter is the porosity of a volume. With a voxel size of 200 μm, Simoneau et al. adjusted the porosity of the structure to reduce the implant stiffness while maintaining strength. The final stem had a porosity of 33%, Young’s modulus of 37 GPa and yield strength of 279 MPa. The smallest unit of stochastic porous structures is often considered as a volume pixel. Compared with the regular porous structure’s unit cell, the volume pixel is generally smaller and more randomly distributed. Hence, it is easier to fill the designated space and more suitable for constructing the femoral stem with complex surface curvature and sharp edges.

## Performance of Porous Femoral Stems

The main factors that determine the long-term survival of a femoral stem are stress shielding, bone ingrowth, and fatigue strength. Therefore, the evaluation of the performance of porous femoral stems is mainly focused on these factors.

### Prevention of Stress Shielding

A key advantage of using a porous structure in the femoral stem is that it can reduce stress shielding by reducing the stiffness of the stem, and thus the ability to avoid stress shielding is an important consideration for evaluating porous femoral stems. The evaluation of stress shielding mainly considers the following aspects:

#### (1) The Stiffness of the Femoral Stem

The greater the stiffness of the femoral stem, the less likely it is to undergo bending deformation *in vivo* and transfer load to the femur. Therefore, reducing the stiffness of the femoral stem is conducive to avoiding stress shielding. Mehboob et al. found that the degree of stress shielding is positively correlated with the axial stiffness of the femoral stem (Mehboob et al., 2020a). Jette et al. calculated the axial stiffness of the femoral stem under axial compressive load according to the force-displacement curve using finite element analysis and *in vitro* mechanical experiments. The results showed that the stiffness of porous Ti6Al4V femoral stems is up to 31% lower than their non-porous counterpart (Jette et al., 2018). Similarly, using a cantilever bending test, Hazlehurst et al. found that the stiffness of a porous CoCrMo alloy femoral stem was up to 60% lower than a traditional solid femoral stem (Hazlehurst et al., 2014a). Mehboob et al. reported that a BCC porous femoral stem with a porosity of 47.3% had a stiffness value closest to that of femoral bone. However, although the stiffness of the femoral stem is closely related to stress shielding, the load transfer from the femoral stem to the femur is not only determined by the stiffness but also by the stem geometry and the fitness to the medullary cavity (Diegel et al., 1989).

#### (2) Stress on Femur

The stress on the femur is the most direct indicator of loading on the femur and has been used in most studies to evaluate stress shielding. While *in vitro* mechanical experiments are an accurate and reliable way of measuring material stress, only the stress values on the surface of the femur can be obtained by strain gauges or digital image correlation technology, and the stress distribution in the interior of the femur can not be measured. Finite element analysis has become an effective method to study the load transmission from the stem to the femur. Using the finite element method, Harrysson et al. evaluated the degree of load transfer from the femoral stem to the femur according to the stress distribution on the femur after implantation of different femoral stems (Harrysson et al., 2008). Hazlehurst et al. marked 25 nodes in the Gruen zone of the femur and evaluated stress shielding at each Gruen zone (Levadnyi et al., 2017) of the proximal femur according to the average von Mises stress on the nodes distributed in each partition (Hazlehurst et al., 2014b). Limmahakhun et al. set up two paths in the medial and lateral femur, respectively, and calculated the average Von Mises stress on each path to evaluate the stress shielding of each Gruen zone (Limmahakhun et al., 2017a). Also, in addition to assessing stress on the femur, stress shielding can also be evaluated according to the stress change ratio of the femur after the implantation of the femoral stem (Eq. 4).

$$Stress\;Shielding\;\left(SS\right)=\frac{S_{\mathrm{int}}-S_{stem}}{S_{\mathrm{int}}}\times 100\%$$ (4)

S_int_ is the average von Mises stress on the intact femur, S_stem_ is the average von Mises stress on the implanted femur.

The relationship between physiological load and bone remodeling has been used to simulate the change in bone mineral density after total hip arthroplasty, which could help evaluate the effectiveness of long-term fixation of porous femoral stems. According to Huiskes’ strain-adaptive bone remodeling theory (Huiskes et al., 1992), the strain energy density (SED) in the bone can be used to quantify stress shielding. The change of bone density ( $\frac{d\rho }{dt}$ ) can be used to describe the process of bone remodeling, according to .

$$\frac{d\rho }{dt}=\{\;\begin{array}{c} <0\\ \;\;\;\;\;0\\ >0 \end{array}for\begin{array}{c} S<\left(1-s\right)\;S_{ref}\\ \left(1-s\right)\;S_{ref}<S<\left(1+s\right)\;S_{ref}\\ S>\left(1+s\right)\;S_{ref} \end{array}$$ (5)

S ( $S=\frac{1}{n}\overset{n}{\underset{i=1}{\sum }}\left(\frac{U_{i}}{\rho }\right)$ ) is the strain energy density (SED) in the femur, where U_i_ is the strain energy in load case i, n is the number of loadings, ρ is the apparent density, S_ref_ is the SED in the intact bone, and s is an empirical constant used to define a dead zone where bone remodeling does not occur. In Huiskes’ study, s was reported to be 0.75 (Huiskes et al., 1992).

In addition, in most studies, in order to reduce the calculation time, the porous section of the femoral stem was often simplified as a solid model, whose material properties were set to be equivalent to the apparent elastic modulus of a porous structure (Hazlehurst et al., 2014b; Limmahakhun et al., 2017a; Arabnejad et al., 2017; Alkhatib et al., 2019a; Mehboob et al., 2020a). Although Jette et al. found that the results of this simplified finite element model were consistent with that of the *in vitro* mechanical experiment (Jette et al., 2018), another study found that the strain field of the simplified model did not match the data measured by digital image correlation technology (Simoneau et al., 2017). Another major limitation of simplifying porous structures to solid material is that it cannot reliably evaluate local stress, and the contact mode between the porous structure and bone at the microscale also changes from surface-to-surface to point-to-face contact. Further investigation is required to understand how changing the contact mode affects the stress on the bone.

A common method for designing porous femoral stems is to simulate bone remodeling after implantation and then afterwards optimize the design of the porous stem structure (Arabnejad et al., 2017; Wang et al., 2018; Wang et al., 2020). However, bone remodeling is also affected by many other factors, such as the shape of the implant, type of surgical operation, and individual differences in anatomy. The long-term effectivity of porous femoral stems needs to be verified through clinical studies before there is confidence in these novel designs. At present, none of the porous femoral stems introduced above have been used in clinical practice.

### Promotion of Bone Ingrowth

Total hip arthroplasty (THA) aims to restore hip joint function by replacing the diseased joint with a prosthesis. The effectiveness of the new motion pair is heavily influenced by the strength of the fixation between the prosthesis and bone. Cementless femoral prostheses undergo fixation in two stages, (i) initial stability achieved through the press-fit between the femoral stem and the medullary cavity and (ii) secondary stability, or long-term fixation, by bone ingrowth onto the stem surface, which is known as osseointegration. Effective bone ingrowth can provide secure fixation between the stem and bone, thus providing good long-term stability and reduce aseptic loosening.

#### (1) Parameters Evaluating Bone Ingrowth

**FIGURE 5:**
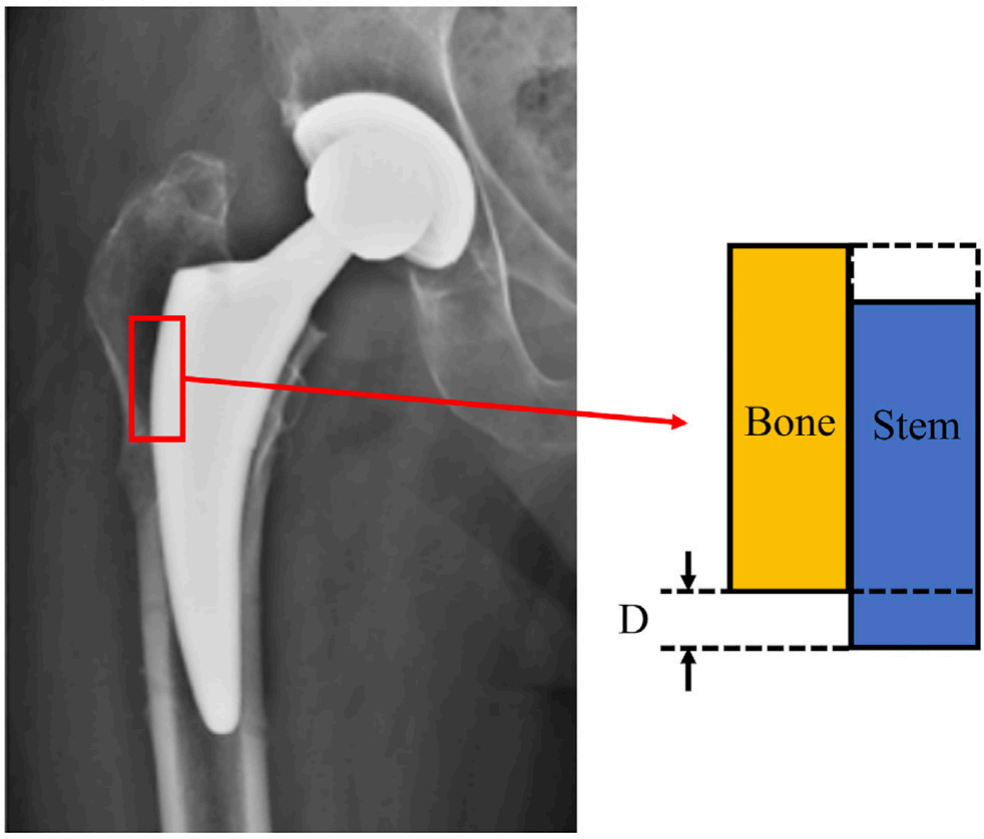
Details of the bone-implant interface, **D** represents interface micro-motion.

Good initial stability of the prosthesis is a prerequisite for bone ingrowth, which is affected by the relative micro-motion between the prosthesis and bone (Büchler et al., 2003) (Figure 5). Previous studies have shown that excessive micro-motion (greater than 150 μm) results in the ingrowth of fibrous tissues into the femoral stem, but is not conducive to the ingrowth of bone tissue. Numerous studies have shown that micro-motion between the femoral stem and bone interface is negatively correlated with the stiffness of the femoral stem (Alkhatib et al., 2019b). The main purpose of designing stems with a porous internal structure is to reduce stiffness to avoid stress shielding of the bone, but the reduced stiffness may also cause excessive interface micro-motion. This point is critical when designing femoral stems, that features designed to reduce stress shielding must be evaluated as to whether they introduce excessive micro motion in the early stages after surgery. As such, Alkhatib et al. found that BBC structures with a porosity greater than 80% can produce excessive interface micro-motion (Alkhatib et al., 2019b). Similarly, Wang et al. constructed a diamond-like porous structure and recommended that the porosity not exceed 50% to keep micro-motion within acceptable limits (Wang et al., 2020). Interface micro-motion can also be controlled by adjusting the stiffness of different regions of the femoral stem (Limmahakhun et al., 2017a; Alkhatib et al., 2019b; Wang et al., 2020), for example by designing a femoral stem with greater stiffness around the proximal end or inner core.

The risk of instability caused by excessive micro-motion can be described by the interface failure index F (b), which should be less than 1 to ensure there is minimal risk of interface failure. As shown in Eq. 6, F (b) is determined by the local shear stress ( τ(b) ) at the bone-implant interface and the bone density ( ρ(b) ) at point b (Wang et al., 2018). This means that the shear stress should not be greater than $21.6\rho {\left(b\right)}^{1.65}$ , with a higher bone density indicating a lower risk of interface failure. Two studies (Arabnejad Khanoki and Pasini, 2013; Wang et al., 2018) used this theory to optimize the design of the porous structure to make sure that the design of the femoral stem does not introduce excessive stress shielding and micro-motion at the bone-implant interface.

$$F\left(b\right)=\frac{\tau \left(b\right)}{21.6\rho {\left(b\right)}^{1.65}}$$ (6)

#### (2) Simulation Study on the Bone Ingrowth

During the healing stage after THA, the surface of the porous stem is initially filled with callus (healing tissue), which will be replaced by bone over time. The ossification process of callus is a complex process affected by the mechanical environment (Isaksson et al., 2006). Some studies have proposed mechano-regulation algorithms using different mechanical stimuli, such as strain, pore pressure, and fluid velocity, as biological stimulation signals to describe this process (Huiskes, 1997; Isaksson et al., 2006; Andreykiv et al., 2008; Tarlochan et al., 2017). Among them, the mechano-regulation theory based on deviatoric strain (DS) proposed by Isaksson et al. (Isaksson et al., 2006) is widely used to predict the osseointegration process at the bone-stem interface (Mehboob et al., 2017; Mehboob et al., 2020a). In Lacroix et al.'s model, the process of tissue differentiation can be divided into six stages based on the deviatoric strain level: granulation tissue, fibrous tissue (DS>5%), cartilage (5%>DS>2.5%), immature bone (2.5%>DS>0.05%), intermediate bone (2.5%>DS>0.05%), and mature bone (DS<0.05%). The Young’s modulus of tissue increases with the level of callus ossification. Therefore, the osseointegration between the callus and the implant becomes stronger with a higher level of differentiation of the callus, resulting in a more effective fixation of the femoral stem.

Using the theory of mechano-regulation algorithms based on deviatoric strain, Mehboob et al. studied how the thickness of the porous surface (determines the callus thickness) and stem stiffness affects bone formation (Mehboob et al., 2020a). The initial stage of the callus around the femoral stem is considered granulation tissue. The principal strains (ε1, ε2, ε3) on each callus element were used to calculate deviatoric strain, as shown in Eq. 7. Thus, tissue differentiation at each iteration process can be predicted according to the principal strains. The results showed that the lower the stiffness of the femoral stem, the higher the degree of tissue differentiation. In addition, the thicker the porous surface, the greater the stiffness of the femoral stem needed to ensure initial stability, thus generating less interface micro-motion and promoting tissue differentiation.

$$DS=\frac{2}{3}\sqrt{{\left(\varepsilon_{1}-\varepsilon_{2}\right)}^{2}-{\left(\varepsilon_{2}-\varepsilon_{3}\right)}^{2}-{\left(\varepsilon_{3}-\varepsilon_{1}\right)}^{2}}$$ (7)

#### (3) *In-vivo* Study on the Bone Ingrowth

**FIGURE 6:**
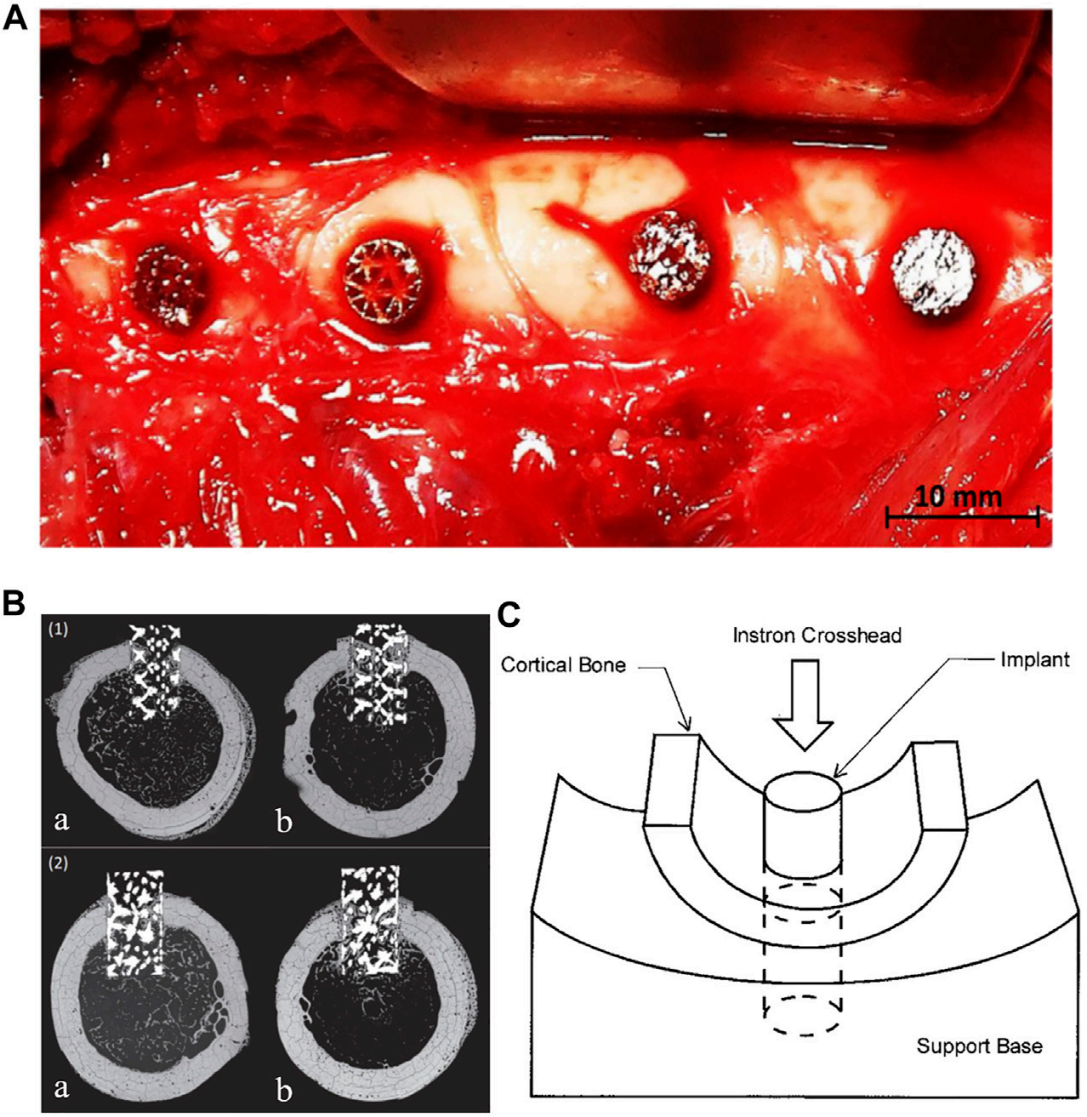
**(A)** Intraoperative photograph of porous implants in the lateral femoral cortex. **(B)** Backscattered scanning electron micrograph of a transverse (1) Octet truss and (2) Tetrahedron transcortical implant section at (a) 4 weeks and (b) 8 weeks. Bone ingrowth can be seen throughout the length of the implant at 8 weeks after surgery (Arabnejad et al., 2016). **(C)** Diagram of push-out test. (Bobyn et al., 1999).

Although mechano-regulation algorithms can indirectly predict bone formation after implantation of a porous femoral stem, the actual process of bone formation is the result of a variety of factors, including the *in vivo* biological environment. At present, to the author’s knowledge, clinical trials have not yet been performed on any femoral stems with internal porous structure, and as such there is no corresponding clinical data available to verify the effectiveness of numerical simulations. However, animal experiments have been used to study bone ingrowth on porous implants. Arabnejad et al. implanted two kinds of porous metal cylinders composed of octet truss and tetrahedron into the femur of dogs (Figures 6–A) and removed the implants after 4 and 8 weeks for histological tests to quantitatively evaluate bone ingrowth (Arabnejad et al., 2016), as shown in Figures 6–B. The results showed that bone ingrowth was positively correlated with the porosity of the implant and the octet truss implant showed more bone ingrowth than the tetrahedron implant.

In Arabnejad et al.’s study, the short porous implants were inserted into holes created with an electric drill in the lateral femoral cortex of dogs. The loading conditions would be considerably different from those on a femoral stem implanted in the medullary cavity. In addition to histological observation, the push-out or pull-out force of the femoral stem could also be used to evaluate the fixation strength after bone ingrowth. As shown in Figures 6–C, Bobyn et al. designed a mechanical experiment to measure the push-out force of a porous implant from the surrounding bone (Bobyn et al., 1999). The load was applied to the end of a cylindrical implant and the peak load recorded. The fixation strength was calculated by dividing the peak load by the cross-sectional area of the cortical bone connected with the implant.

### Prevention of Fatigue Fracture

The load acting on the hip joint in a gait cycle is about 2.5–3 times body weight. After the femoral stem is implanted into the body, it not only has to bear the bodyweight but also have sufficient fatigue life to withstand the repeated loading during daily activities. In addition to the stiffness of porous stems typically being lower than solid ones, the fatigue strength is also often lower because they are made using additive manufacturing technology (Amin Yavari et al., 2013). Therefore, the fatigue strength should be carefully evaluated when designing porous femoral stems. Mechanical tests and fatigue analyses are commonly used methods for evaluating and optimizing the fatigue durability.

#### (1) Mechanical Test Method

Fatigue testing according to ISO 7206-4:2010 (Standardization, I.O.f., and ISO7206-4, 2010) is the most common approach for evaluating the strength of a porous femoral stem. This test requires the distal end of the femoral stem to be fixed in an embedding medium (usually bone cement), and the stem body aligned with 10° anteversion and 9° abduction. The head of the femoral stem is then subjected to a vertical downward cyclic load ranging from 300 to 2600 N for 5×10^6^ loading cycles. Yang et al. (2009) designed three types of hollow femoral stems, which are a stem with round holes in the proximal region, a stem with long holes in the proximal region, and a stem with both round holes and long holes in the proximal region. Fatigue tests were performed on all three femoral stems in accordance with ISO 7206-4:2010, and accompanied by finite element (FE) models to simulate the fatigue test. The three femoral stems survived the full 5 × 106 loading cycles, with the FE models showing the areas most prone to fatigue fracture (showing high stress concentration) being mainly concentrated around the middle lateral region of the stem and the regions around the pores. Due to the high stresses observed around the pores in the femoral stem, fatigue cracking is more likely to occur in this area (Zhou and Soboyejo, 2004). These cracks may expand due to corrosion from the fluid medium, so the porous structure of the femoral stem is more prone to fatigue failure (Yang et al., 2009; Arabnejad Khanoki and Pasini, 2013). However, ISO 7206–4 does not specify the fluid medium for testing and the choice of fluid medium may affect the results of the fatigue test. In addition, the mean hammering force when a surgeon inserts the femoral stem into the femur using a hammering is reported to be 9.2 kg (Sakai et al., 2011), which is over ten times the body weight (assuming to be 75 kg). Although most porous femoral stems are designed with solid femoral necks to meet the strength requirements and fit with a standard femoral head, however, when the stem is implanted, excessive percussion forces may fracture the porous structure. Further testing may be required to verify if the femoral stem can meet the strength requirements during implantation.

#### (2) Finite Element Method

Fatigue testing using a finite element model is another approach to assessing fatigue life, such as the Soderberg fatigue theory and Goodman fatigue theory. Although the Soderberg theory is more conservative, studies have shown it to be accurate in predicting the fatigue life of porous femoral stems (Yang et al., 2009; Arabnejad Khanoki and Pasini, 2013; Mehboob et al., 2020b). Using this method, the maximum stress ( $\sigma_{\max}$ ) and minimum stress ( $\sigma_{\min}$ ) generated in the porous femoral stem were used to calculate mean stresses (σ_m_) and alternating stresses (σ_a_) in one load cycle, as defined by Eq. 8, 9.

$$\sigma_{m}=\frac{\left(\sigma_{\max}+\sigma_{\min}\right)}{2}$$ (8)

$$\sigma_{a}=\frac{\left(\sigma_{\max}-\sigma_{\min}\right)}{2}$$ (9)

According to the Soderberg approach, the femoral stem will not undergo fatigue failure if $\sigma_{\max}$ and $\sigma_{\min}$ are both located below the Soderberg line, defined as follows:

$$\left(\frac{\sigma_{a}}{S_{e}}\right)+\left(\frac{\sigma_{m}}{S_{ys}}\right)=\frac{1}{N}$$ (10)

Where S_e_ is the endurance limit of the material, which can be estimated according to the S/N curve of the material (Senalp et al., 2007). S_ys_ is the yield strength of the material, and N is the factor of safety.

Arabnejad et al. integrated the Soderberg fatigue theory into the design of a porous femoral stem (Arabnejad Khanoki and Pasini, 2013). They proposed applying smoother geometric unit cells or designing a solid core in the femoral stem to improve the fatigue strength.

### Materials

Femoral stems must be able to withstand long-term cyclic mechanical loading and the corrosive effects of body fluids, so there are strict requirements around biocompatibility, safety, and effectivity. At present, porous femoral stems manufactured through 3D printing are mainly made of titanium alloy (Ti6Al4V) or CoCrMo alloy (Co-Cr-Mo), as shown in Table 2.

**TABLE 2:** Materials and manufacturing methods of porous femoral stems.

No	Material	Approach	Equipment	Laser spot diameter (μm)	Powder size (μm)	Powder layer thickness (μm)	References
1	Ti6AL4V	EBM	-	-	45–100	-	Arabnejad Khanoki and Pasini, (2013)
2	Ti6AL4V	SLM	Renishaw AM250 (Renishaw Limited, Mississauga, ON)	70	15–50	30	Arabnejad et al. (2017)
3	Ti6AL4V	EBM	Arcam AB (Arcam, Molndal, Sweden)	-	-	-	Yang et al. (2009)
4	Ti6AL4V	EBM	Arcam Q10 (Arcam, Molndal, Sweden)	-	45–105	50	Eldesouky et al. (2017b)
5	Ti6AL4V	EBM	Arcam A2 (Arcam, Molndal, Sweden)	-	-	-	Eldesouky et al. (2017a)
6	Ti6AL4V	EBM	-	-	-	-	Harrysson et al. (2008)
7	Ti6AL4V	SLM	EOS M280 (EOS GmbH, Munich, Germany)	-	-	30	Mehboob et al. (2020b)
8	CoCrMo	SLM	EOS M270 (EOS GmbH, Munich, Germany)	100	≤63	20	Hazlehurst et al. (2014a)
9	Ti64	SLM	EOS M280 (EOS GmbH, Munich, Germany)	-	-	-	Jette et al. (2018)
10	CoCr	SLM	-	-	-	100	Limmahakhun et al. (2017a)
11	Ti64	SLM	EOS M280 (EOS GmbH, Munich, Germany)	-	-	30	Simoneau et al. (2017)
12	Ti6AL4V	SLM	EOS M290 (EOS GmbH, Munich, Germany)	-	4–55	-	Abate et al. (2021)
13	Ti6AL4V	SLM	Laseradd DiMetal-280 (Laseradd, Guangzhou, China)	-	-	-	Wang et al. (2018)

Porous femoral stems made of Ti6Al4V have been reported with good biocompatibility and corrosion resistance (Ryan et al., 2008; Arabnejad et al., 2017; Mehboob et al., 2020b). And compared with 316L stainless steel (210 GPa) and CoCrMo alloy (240 GPa), Ti6Al4V has a lower Young’s modulus (55–110 GPa) (Zhang and Attar, 2016) and higher strength to weight ratio (Head et al., 1995). Arabnejad et al. fabricated Ti6Al4V porous femoral stem with an average pore size of 500 μm. Through mechanical experiments, they found that the porous femoral stem can significantly reduce the stress shielding on the medial end of the proximal femur by comparison with the solid one in the case of the same material (Arabnejad et al., 2017). Considering that aluminum has been linked to the development of Alzheimer’s disease and vanadium is cytotoxic (Ryan et al., 2008; Sidhu et al., 2020), titanium alloys have been developed without these materials, such as Ti35Nb7Zr5Ta (TNZT), Ti12Mo6Zr2Fe (TMZF) and Ti32Nb8Zr4Ta, which could potentially be used for manufacturing porous femoral stems (Eldesouky et al., 2017a; Sidhu, 2021). Eldesouky et al. found that TNZT and TMZF have a lower stiffness (55–85 GPa) than Ti6Al4V and produce less stress shielding (Eldesouky et al., 2017a). Sidhu et al. used electrical discharge machining (EDM) on a Ti32Nb8Zr4Ta implant to obtain a biocompatible porous surface that could better promote bone ingrowth (Sidhu, 2021).

Another option is to manufacture femoral stems from CoCrMo alloy (Hazlehurst et al., 2014a; Limmahakhun et al., 2017a). CoCrMo has better wear resistance, corrosion resistance and ultimate strength than titanium alloy (Milošev, 2012) and is cheaper to manufacture with, but the stiffness is almost double and so is likely to produce greater stress shielding. However, including a porous inner structure in the femoral stem would lower the stiffness and could make this alloy a viable option. Alternatively, a monoblock CoCrMo porous femoral stem was developed by Hazlehurst et al. and shown to have a stiffness nearly 60% lower than a fully dense stem, which helps avoid stress shielding (Hazlehurst et al., 2014a). The integrated design also reduces the production of wear particles between the head-neck taper. Limmahakun et al. also successfully fabricated a CoCrMo porous femoral stem via AM. The stem had a mass of approximately half of the solid stem, and its flexure stiffness was about one-tenth that of the solid stem (Limmahakhun et al., 2017a). However, the studies above only fabricated the femoral stem (excluding the femoral neck and femoral head) and carried out three-point bending tests. To truly assess the feasibility of these designs, a full prosthesis would be required and tested under physiological loading. In addition, it has been found that the addition of calcium phosphate (CAP) powder during the additive manufacturing process of CoCrMo alloy can form CoCrMo-CaP composites. Compared with CoCrMo alloy without calcium, CoCrMo-CaP composite can significantly improve the wear resistance and is expected to be used for femoral stem (Sahasrabudhe et al., 2018).

Porous tantalum is another potential material for the manufacture of porous femoral stems. Porous tantalum has been reported to have good biological adaptability, good corrosion resistance, low elastic modulus and an excellent ability for osseointegration (Bobyn et al., 1999). In addition, its high friction coefficient can increase the initial stability of the prosthesis (Biemond et al., 2011). Tantalum has primarily been used in artificial hip joints to manufacture the acetabular cup (Unger et al., 2005; Levine et al., 2006). In addition, tantalum has been used to fabricate augments in the treatment of pelvic discontinuity (Sporer et al., 2005). However, to date no porous tantalum femoral stem has been fabricated via AM and so more research is needed in this area.

### Manufacture

It is challenging to prepare complex and interconnected porous structures using traditional manufacturing methods such as by powder metallurgy or powder sintering, and the application of these technologies is limited in terms of the ability to adjust the morphology and distribution of the pores. Additive Manufacturing (AM), first appeared in its basic form in the 1980s where materials were stacked layer by layer to form the required three-dimensional entity (Melican et al., 2001). This bottom-up approach makes it possible to prepare complex porous structures and makes it easier for researchers to design customized implants. Among the various 3D printing technologies, Selective Laser Melting (SLM) and Electron Beam Melting (EBM) are the most widely used to manufacture porous femoral stems. The manufacturing details of the porous femoral stems are shown in Table 2.

Electron beam melting (EBM) uses high-energy and high-speed electron beams to selectively bombard metal powder in a vacuum environment. The metal powder is melted together and is bonded with the formed part, and stacked layer by layer until the whole part is melted. Compared with SLM, EBM allows for a higher molding density, quicker manufacturing times, and lower residual stress. The disadvantage is that the size of the powder bed limits the size of the formed sample, and EBM cannot so far print non-metallic materials such as plastics or ceramics. EBM technology can also use electron beam scanning to preheat each layer of metal powder to reduce the residual stress of the molded parts. After preheating, the metal powder achieves a state similar to pseudo sintering, but the removal of these powders needs to be considered post-treatment.

Selective laser melting (SLM) technology can selectively melt metal powder on a powder bed by controlling the laser beam with a specific wavelength and intensity in an inert gas atmosphere. Compared with EBM, SLM has a smaller beam spot, which is more conducive to forming fine features and complex parts and results in a higher surface quality and mechanical strength. The disadvantage of SLM is that it can produce high residual stress within the finished part.

When using EBM or SLM to manufacture a porous femoral stem, residual metal powder is often found in the pores. Although these powders will not significantly impact the structure’s mechanical properties, they can cause a high level of metal ions in the blood and related complications after total hip arthroplasty (Levine et al., 2013; Chang and Haddad, 2019). Conventional methods for removing the powder include a vibration table or using compressed air (Simoneau et al., 2017; Jette et al., 2018). Harrysson et al. found that by reducing the preheating of the powder bed when using EBM the amount of sintering powder could also be reduced, which made it easier to remove residual melted powder from the pores (Harrysson et al., 2008). Similarly, Eldesouky et al. fabricated porous scaffolds with good geometric consistency using EBM (Eldesouky et al., 2017b), but found an inconsistency between the measured mass and the designed mass, which may be related to the titanium powder retained in the pores.

**FIGURE 7:**
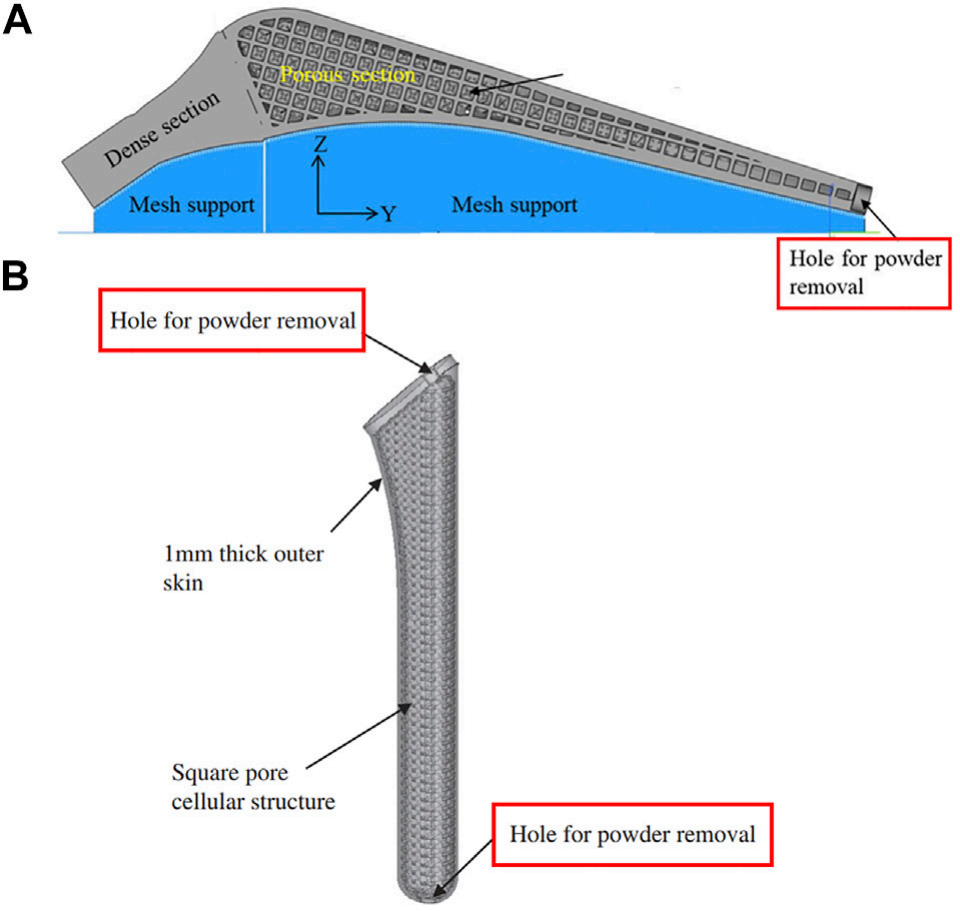
**(A)** Hole positioned at the bottom of the femoral stem for powder removal designed by Mehboob et al. (2020b); **(B)** Powder removal holes at the top and bottom of the femoral stem designed by Hazlehurst et al. (2014a).

Good permeability of the porous structure is helpful when removing residual powder. Jette et al. (2018) and Simoneau et al. (2017) fabricated femoral stems with a regular porous structure and random porous structure via SLM, respectively. The volume of residual powder in the regular porous structure accounted for 3% of the porous volume, which was much less than the 15.5% for the random structure. This was due to the higher tortuosity inside the structure, which made the powder more difficult to remove. In addition to structure permeability, when designing a femoral stem, holes or channels can also be added to help remove residual powder (Mehboob et al., 2020b) (Hazlehurst et al., 2014a) ((Figures 7–A, Figures 7–B). .

**FIGURE 8:**
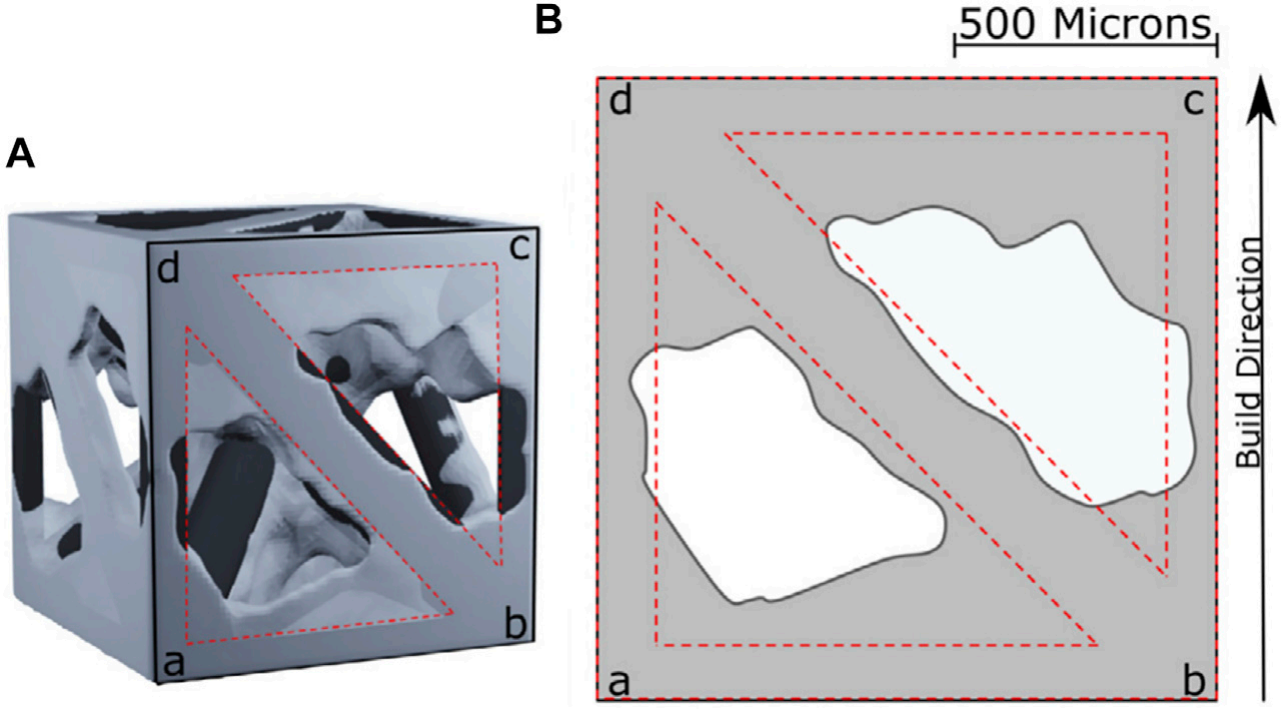
**(A)** 3D reconstruction of a unit cell. **(B)** Discrepancy between design parameters and manufactured parameters (Arabnejad et al., 2016).

A key observation reported in previous studies on porous structures is that the size of the pores after manufacture may be different from the design, which may affect the mechanical properties and osseointegration potential of the implant, as shown in Figure 8. Arabnejad et al. printed a porous femoral stem using EBM with cell sizes of 1, 2, and 3 mm and found that the larger the cell size, the closer the fabricated parameters were to the design parameters (Arabnejad Khanoki and Pasini, 2013). The implant with a cell size of 1 mm had thicker walls (33.5% thicker) and smaller pores (53.6% smaller) than the design, and the pores contained partially melted powder. However, increasing the designed pore sizes to 2 and 3 mm considerably reduced the discrepancy to only 5.5 and 0.1%, respectively. However, large unit cells may not be suitable for bone growth, but coating the material in a conductive layer could potentially resolve this.

The porosity and strut angle unit cells are also important factors affecting manufacturing accuracy. Arabnejad et al. found that the greater the porosity the greater the error between the measured porosity and the designed porosity, and the smaller the angle between the strut and the horizontal plane, the greater the error between the actual strut thickness and the design value (Arabnejad et al., 2016). Acid etching or electropolishing can be used after manufacture to reduce the discrepancy, but this may affect the final mechanical properties of the porous structure (Pyka et al., 2012).

## Other Factors Affecting the Mechanical Behavior of Porous Femoral Stems

In addition to the type of porous structure, the distribution of pores and the shape of the femoral stem are also important factors that may influence the performance. The following will introduce the factors to be considered when designing the macro-morphology of a porous femoral stem.

### Functionally Graded Femoral Stem

**FIGURE 9:**
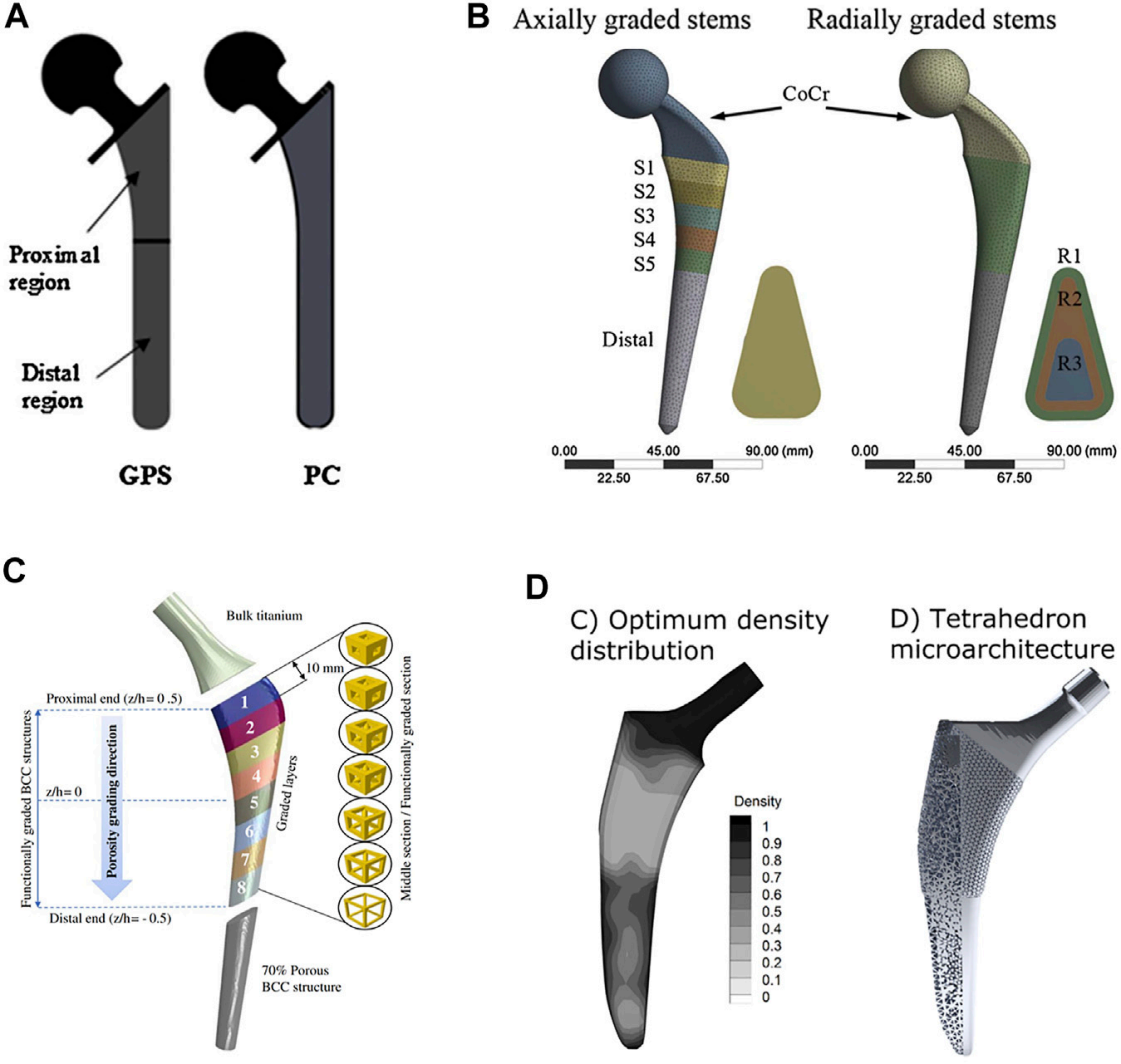
Four types of functionally graded femoral stems: **(A)** A two-step gradient stem designed by Hazlehurst et al. GPS: porous stem with axially graded stiffness, PC: porous stem with radial graded stiffness (Hazlehurst et al., 2014b); **(B)** Multi-level gradient stem designed by Limmahakhun et al. (2017a); **(C)** Smooth gradient stem designed according to a sigmoid function designed by Alkhatib et al. (2019b); **(D)** Stem using an optimum distribution of relative density to minimize stress shielding, designed by Arabnejad et al. (2017).

Functionally graded materials (FGM) are composite materials composed of two or more materials with continuous gradient changes in composition and structure. Local material properties of FGM can be controlled by changing the composition and structure of the materials. The advantage of using FGM in femoral stems is that the properties of specific regions of the stem can be tailored to reduce the stiffness mismatch between the prosthesis and surrounding bone and provide a more uniform stress distribution in the femur. In 2014, Hazlehurst et al. (Hazlehurst et al., 2014b) first used porous materials to achieve a functional gradient in a femoral stem, they designed two femoral stems with axial and radial orientation, respectively. The former was realized by arranging the porous structures with different stiffness at the proximal and distal ends of the femoral stem, while the radial gradient was achieved by having a porous inner core of the stem surrounded by an outer dense metal shell. The results showed that the axial gradient design was better capable of reducing stress shielding in proximal–medial femur than a homogeneous porous structure. However, the radial gradient design with the external dense metal shell did not help to reduce stress shielding. In a related study, Limmahakhun et al. designed graded porous femoral stems with axial and radial orientations incorporating more graded porous sections (Limmahakhun et al., 2017a). The results showed that the axially graded stem with a stiffer proximal end or the radially graded stem with a stiffer inner core performed better at reducing stress shielding and also produced less micro-motion. Alkhatib et al. (2019b) proposed an axially graded femoral stem according to a sigmoid function that had a smooth distribution of pores along the stem’s axis. The porosity of this femoral stem increased gradually from top to bottom, and the porosity changed according to the sigmoid equation. The larger the grading exponents of the sigmoid equation, the more obvious the change in porosity in the upper and lower sections of the femoral stem, and the more gradual the change in porosity around the middle section. However, Alkhatib’s study found that the graded femoral stem with higher stiffness at the proximal end could better reduce bone-implant interface micro-motion than a homogeneous porous femoral stem, and the micro-motion decreased as the grading exponents increased. Arabnejad et al. established an optimization program to minimize stress shielding by adjusting the local density of the femoral stem (Arabnejad et al., 2017). In theory, this optimized femoral stem should be better able to reduce stress shielding than the axial or radial functional gradient femoral stem, and may be better suited to customized prosthesis. Future work may consider customizing the design according to the patient’s weight, bone density, medullary cavity shape or other factors. The four types of functionally graded femoral stems described above are shown in Figure 9.

### Stem Length

According to Feyen’s classification of femoral stem length (Feyen and Shimmin, 2014), a femoral stem whose length is less than twice the vertical distance from the tip of the greater trochanter to the base of the lesser trochanter is defined as a short stem, and those having a greater length are defined as a standard stem. The length and shape of the femoral stem determines the fixation mode against the femoral medullary cavity. Because the cortical bone at the diaphysis is hard and strong, the diaphyseal fixation of the femoral stem often has good initial stability. However, diaphyseal fixation also induces more load transfer to the distal femur, which causes stress shielding in the proximal femur. The fixation of a short stem mainly depends on ‘fit and fill’ with the metaphyseal region, which can transfer more load to the proximal end to avoid stress shielding at this location. Although numerous studies have investigated the effect of the stem length on stress shielding, it is still unclear whether short stems or standard stems are more suitable for stems with a porous structure.

This review classified various porous femoral stems from previous studies according to Feyen’s classification system (Feyen and Shimmin, 2014). The results showed that the number of studies focusing on standard femoral stems was twice that of short stems. Most studies did not consider the effect of stem length on the performance of the porous femoral stem under investigation. Some reports studied the effect of the stem length on stress shielding. However, the incorporation of a porous structure changes the local stiffness of the femoral stem and the stress distribution on the prosthesis and bone. In this case, the effect of the stem length on stress shielding needs to be investigated further. We also found that about half of the porous femoral stems were designed based on the existing commercially available prostheses, while the other half was innovative. The geometric profiles of these innovative femoral stems mostly do not conform to the basic design principles required for a successful femoral prosthesis. For example, some had sharp edges, which might cause excessive local stress concentration on the femur, and others had an overly-thin stem body which may not provide sufficient strength for a press-fit fixation in the femoral medullary cavity.

### Other Design Concepts

**FIGURE 10:**
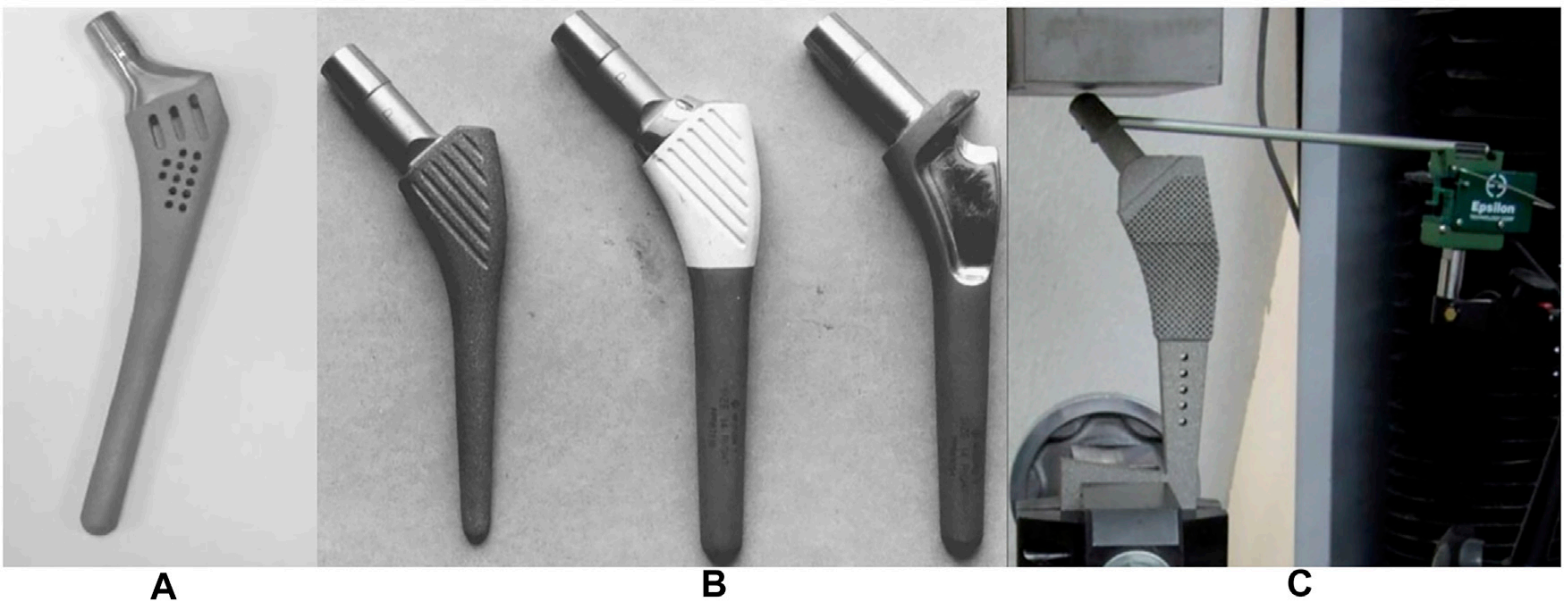
**(A)** hollow femoral stem designed by Yang et al. (2009). **(B)** cement-locked uncemented (CLU) femoral stem (Viceconti et al., 2001). **(C)** cementless stem with porous structure for injecting bone cement, designed by Eldesouky et al. (2017b).

Standard practice when implanting a femoral stem is to hollow out the proximal region of the femur. However, a reduction in intramedullary blood supply may increase the resorption of cortical bone (Pazzaglia, 1996). To compensate, Yang et al. designed a hollow femoral stem with holes on the surface which provide space for medullary revascularization (Yang et al., 2009) (Figures 10–A). The stiffness of the femoral stem was also reduced due to the removal of the material inside the stem, which helped to reduce the level of stress shielding. This porous stem also allowed for bone ingrowth. However, the diameter of the hole was set to be 2 mm, which was much larger than the pore size reported to be suitable for bone ingrowth (50–800 μm) (Harrysson et al., 2008; Arabnejad et al., 2016).

Another noteworthy design concept is cement-locked uncemented (CLU) prostheses, which were first introduced by Viceconti et al. (Figures 10–B) in 2001. With this system, the femoral stem and medullary cavity are connected by a hybrid fixation system, and the initial stability of the femoral stem is achieved by injecting bone cement into pockets located on the lateral part of the femoral stem (Viceconti et al., 2001). However, the presence of the injected cement decreases the area for bone ingrowth. Similarly, Eldesouky et al. designed a porous femoral stem composed of square unit cells at the proximal end which was stabilized after implantation by injecting biodegradable bone cement into the pores (Eldesouky et al., 2017b) (Figures 10–C). In addition, the porous structure at the proximal end and the holes at the distal end of the femoral stem can also reduce the stem stiffness, thereby reducing the stress shielding. Although the porous structure increases the contact area with bone cement and achieves a stronger stem-cement bond, due to differences in elasticity of the femoral stem, cement shell and bone, and the cyclic load acting on the hip joint, interface micro-motion between the stem and cement is inevitable (Cristofolini et al., 2003; Scheerlinck, 2011). The rougher the femoral stem interface is, the easier it is to damage the bone cement shell and produce cement debris, which may cause osteolysis (Verdonschot, 1998). And owing to the smaller cross-sectional area of the cemented femoral stem than the uncemented stem, the bending stiffness of the cemented femoral stem is lower, and it produces less stress shielding.

## 6 Summary and Future Direction

### Summary

This paper reviewed previous research into porous femoral stems, including the structural/morphological characteristics of the porous structure, the mechanical and biological performance of the femoral stem, factors affecting performance, and material and manufacturing techniques.

1) As for the porous structure applied to the femoral stem, most previous studies used regular porous structure types. BCC, tetrahedral and diamond lattices are porous lattice structures that are more widely used in femoral stem because of their good isotropy, low stiffness and high strength. Furthermore, previous studies aimed to optimize the design parameters and distribution of the porous structures in the femoral stem according to various mechanical and biological factors (stress shielding, interface micro-motion, fatigue strength, and bone ingrowth).
2) Most published data on bone ingrowth into porous femoral stems were estimated indirectly by measuring interface micro-motion or through bone ingrowth simulations by FEA. In addition, effects of muscle tissues on the bone-stem mechanical transmission were ignored by many studies. A small number of studies inserted porous cylindrical implants in animals to investigate the *in vivo* performance, but such models may not be representative of human femoral bone under different loading conditions.
3) The macro design of a femoral stem, including stem length and the presence of a collar, are factors affecting the mechanical performance, but were not considered in most previous studies of porous femoral stems.
4) The most common materials used for porous femoral stem are Ti6Al4V and CoCrMo alloys. Alternatively, novel materials (Ti35Nb7Zr5Ta (TNTZ) and Ti12Mo6Zr2Fe (TMZF)) which present lower stiffness and cytotoxicity than traditional Ti6Al4V, have shown a potential application in the porous femoral stems. However, there is no manufacture case of the femoral stem using the above materials.

### Future Direction

1) When designing a porous femoral stem, not only do the parameters referring to the stiffness, strength, isotropy, and potential for bone ingrowth need to be considered, but the distribution of pores is also important. The pores in the femoral stem could be arranged according to the stress distribution on the stem for the purpose of achieving the best mechanical performance.
2) Metamaterials, such as an auxetic structure, have shown potential application in the femoral stem, thus a combination of several types of porous structures could be used to tailor the various mechanical and biological requirements along the femoral stem.
3) A key advantage of additive manufacturing is the ability to easily manufacture customized implants to fit an individual patient’s needs. A customized femoral stem with optimal mechanical and biological adaptation characteristics according to the weight, bone density, and morphology of the medullary cavity may be recommended in the future. In addition, additive manufacturing is also helpful to design the femoral stem with powder removal holes to help remove any residual powder after manufacture.
4) The design of porous femoral stem needs to consider the practicality for clinical applications, avoiding some stems being too thin or having sharp edges. Moreover, if good bone ingrowth into the porous femoral stem is achieved, how to remove the stem in the revision surgery is something that also needs to be considered.
